# The cross-talk and interplay between ferroptosis and cuproptosis in tumor and therapeutics

**DOI:** 10.1007/s13402-025-01138-6

**Published:** 2026-01-08

**Authors:** Xiangting Zhou, Shuping Peng

**Affiliations:** 1 NHC Key Laboratory of Carcinogenesis, Cancer Research Institute, Xiangya School of Basic Medical Sciences, Central South University, Changsha, Hunan 410013 China; 2 The Key Laboratory of Carcinogenesis and Cancer Invasion of the Chinese Ministry of Education of Xiangya Hospital, Central South University, Changsha, 410078 China; 3 FuRong Laboratory, Changsha, Hunan 410078 China

**Keywords:** Ferroptosis, Cuproptosis, Cellular metabolism, Crosstalk, Interplay, Tumor progression

## Abstract

Ferroptosis and cuproptosis are two recently discovered forms of programmed cell death. Both forms of cell death are regulated by distinct yet interconnected pathways, and their roles in tumor progression and therapy response have gained much concern and are highly anticipated. Both involve alterations in cellular metabolism. Oxidative stress plays a crucial part in both processes. Ferroptosis mainly involves the peroxidation of cell membrane lipid components, while cuproptosis is more related to mitochondrial dysfunction and the stability of iron sulfur cluster proteins. Moreover, ferroptosis is usually related to iron and antioxidant capacity, while cuproptosis focuses more on the imbalance of copper homeostasis and its impact on organelles. The increase in reactive oxygen species can trigger DNA damage and other cellular stresses that impact tumor development and response to treatment. In ferroptosis, iron catalyzes the Fenton reaction, generating hydroxyl radicals that cause oxidative damage. Copper metabolism also intersects with iron metabolism. For example, copper chaperones such as copper chaperone for superoxide dismutase regulate intracellular copper levels and indirectly affect iron handling. Crosstalk between ferroptosis and cuproptosis involves diverse signaling pathways. In this review, we recapitulate the way these two sorts of programmed cell death interact, providing insights into mechanisms of therapeutic resistance. Targeting both pathways simultaneously or sequentially may well surmount resistance and boost treatment efficacy. In summary, the shared mechanisms and interplay between ferroptosis and cuproptosis offer exciting opportunities for enhancing our knowledge of tumor biology and improving cancer treatment paradigms. Continued research in this area promises to uncover new targets and strategies for treatment of cancer.

## Clinical trial number

Not applicable.

## Introduction

Tumors represent a complex malady severely threatening human health, with their pathogenesis entailing the combined involvement of environmental and genetic factors [1]. Cell death acts as a crucial regulatory approach for upholding cellular homeostasis. It plays an essential part in maintaining the typical physiological operations of cells, and is also involved in the origin and evolution of diseases such as tumors, as well as the generation of treatment resistance [2]. Resistance to cell death marks a vital biological property of tumor cells, which will lead to the proliferation and activation of tumor cells or enable them to evade the killing effects of treatment approaches such as drugs and radiation on tumor cells [3, 4]. Cell death represents a complex process unfolding in multiple ways and can be classified into different types according to its occurrence modes and regulatory mechanisms. Traditionally, it is classified into programmed cell death and non-programmed cell death. Programmed cell death encompasses apoptosis, autophagy, necroptosis, pyroptosis, ferroptosis, and the recently discovered copper-dependent cell death, namely cuproptosis [5, 6].

**Fig. 1:**
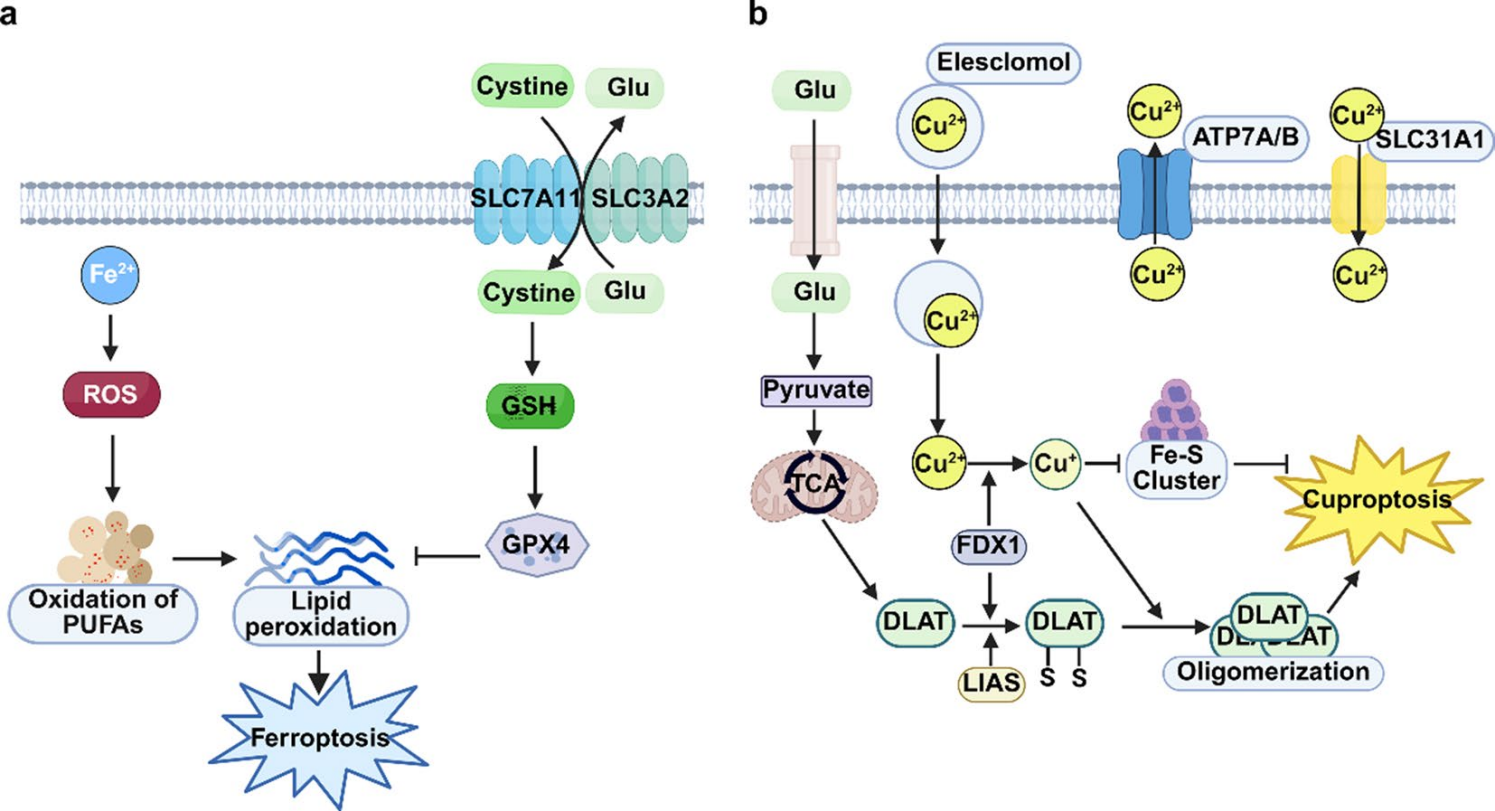
Regulatory mechanism of ferroptosis and cuproptosis. (**a**) Molecular mechanism of ferroptosis; (**b**) Molecular mechanism of cuproptosis. (Created with Biorender.com )

Ferroptosis and cuproptosis are two novel forms of programmed cell death, both of which are metal ion-dependent cell death. In 2012, Brent Stockwell first discovered ferroptosis, a type of cell death. It is marked by an abnormally increased intracellular iron content and the accumulation of reactive oxygen species (ROS), which further causes lipid peroxidation and finally leads to cell death. The classic ferroptosis pathways are mainly regulated in two ways: first, the accumulation of intracellular free Fe²⁺ generates ROS through the Fenton reaction. These ROS continuously oxidize polyunsaturated fatty acids (PUFAs) within membrane phospholipids, leading to the excessive accumulation of lipid peroxides and the disruption of membrane integrity, ultimately triggering cell death. Second, ferroptosis is also regulated by the cystine/glutamate antiporter System Xc⁻, a dimeric protein complex composed of two subunits, solute carrier family 3 member 2 (SLC3A2) and solute carrier family 7 member 11 (SLC7A11). The SLC7A11 subunit is responsible for transporting cystine into cells for the synthesis of glutathione (GSH). As the major intracellular antioxidant, GSH acts as a substrate for glutathione peroxidase 4 (GPX4), which removes intracellular peroxides and maintains redox homeostasis. Therefore, when SLC7A11 expression is inhibited, GSH synthesis decreases and GPX4 activity is reduced, thereby increasing the cell’s susceptibility to ferroptosis or directly inducing its occurrence [7]. (Fig. 1a). Cuproptosis was recently discovered by Peter Tsvetkov in 2022. In case of excessive intracellular copper ions, they will directly bind to lipoylated proteins in the mitochondrial tricarboxylic acid (TCA) cycle. As a result, these proteins will abnormally aggregate, and iron-sulfur cluster proteins will be exhausted. Subsequently, this triggers a proteotoxic stress response and finally drives the cells to death [6] (Fig. 1b).

Iron and copper are vital trace elements within the human body, having crucial roles in many physiological processes and the onset of diseases. Both rely on the excessive accumulation of metal ions, which leads to the aggregation of intracellular peroxides and cellular stress, and then induces cell death. In the past few years, studies have indicated that there is mutual regulation between these two metal ions in the course of inducing cell death. That is, copper ions may regulate iron homeostasis, and iron ions may also inhibit copper metabolism [8, 9]. Meanwhile, there is also certain cross–regulation among the regulation of intracellular iron ions’ and copper ions’ homeostasis and the relevant signal pathways for the occurrence of ferroptosis and cuproptosis. Therefore, cuproptosis and ferroptosis may not be completely independent events within a cell, and there is a certain association between them. This article has summarized the hallmarks of ferroptosis and cuproptosis, the regulation of metal ion homeostasis, the associations among signal pathways, and their roles in tumor progression and treatment resistance.

## Metal ions have mutual regulatory effects during the processes of cuproptosis and ferroptosis

### Mutual regulation exists in the homeostasis of iron ions and copper ions

Iron and copper have similar physical and chemical properties, which determines that there may be physiologically relevant interactions between copper and iron. An escalating number of studies have revealed that, on the one hand, copper can antagonize iron metabolism, and on the other hand, it can also affect the maintenance of iron homeostasis. When the iron reserve in the body is low, copper can be redistributed to tissues that play important roles in regulating iron balance, including the enterocytes in the upper segment of the small intestine, the liver and the blood, thus promoting the transportation or release of iron. For example, copper in enterocytes may have a positive impact on iron transportation, and copper in the liver may promote the biosynthesis of the circulating ferroxidase ceruloplasmin, thereby facilitating the release of iron from storage. Meanwhile, in the case of iron deficiency, copper can modulate the DNA-binding activity of HIF-2α, thus promoting the transcriptional activity and expression of a series of intestinal genes in connection with iron absorption and the maintenance of intestinal iron homeostasis. Iron regulates HIF-1α stability via Fe²⁺-dependent prolyl hydroxylases (PHDs) [10], whereas copper influences its activity through redox- and signaling-mediated mechanisms [11], thereby linking metal homeostasis to angiogenesis and tumor progression. Conversely, too many iron ions can also restrain the utilization of copper ions [12, 13]. For example, a large amount of iron supplements taken may lead to copper deficiency [14, 15]. Therefore, based on the above-mentioned homeostatic interactions, copper and iron do not independently induce cell death under certain conditions; instead, they can mutually trigger or amplify each other’s lethal signals.

### Mutual regulatory effects of iron ions and copper ions on the induction of ferroptosis and cuproptosis.

#### Copper-ion overload can induce ferroptosis

As a trace element, copper is of fundamental significance to human physiological processes, and changes in its metabolism are tightly linked to the occurrence of various diseases. In patients with malignant tumors, such as lymphoma, breast cancer, and lung cancer, the copper levels in serum and tumor tissues rise significantly. Research has proven that copper ions have a multifaceted function in tumor progression. First, the elevated copper levels in tumors fuel tumor growth. This is probably because tumor cells have a higher need for copper-related metabolic activities. For example, many copper-dependent enzymes are over-activated in renal cell carcinomas (RCC), promoting rapid cell division [16]. Conversely, copper ion chelation significantly impedes tumor growth. By chelating copper ions, the amount of free copper ions in tumor cells is decreased. As a result, copper-dependent enzymes essential for tumor cell survival, multiplication, and angiogenesis, along with relevant signaling pathways, are disrupted [17, 18]. This dua-nature of copper ions in tumors shows that copper ions and their metabolic processes are intricately linked to tumorigenesis and tumor development.

**Fig. 2:**
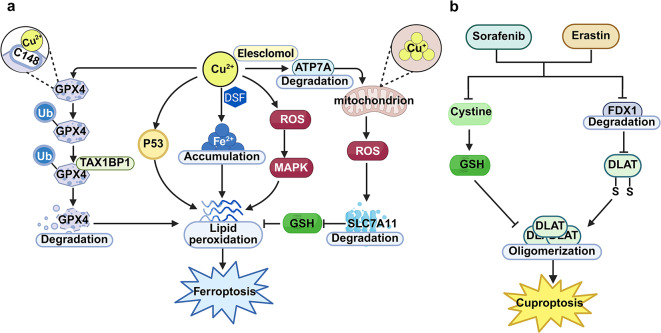
The mechanism of ferroptosis induced by copper ions and of cuproptosis induced by ferroptosis inducers. a. The mechanism of ferroptosis induced by copper ions; b. The mechanism of cuproptosis induced by ferroptosis inducers (Created with Biorender.com)

Recent investigations have revealed that ferroptosis is also an important pathway for copper-triggered cell death, and it is highly significant in tumorigenesis and malignant progression. On the one hand, an overload of copper ions can induce changes in iron ion concentration and the expression of ferroptosis-related markers. For example, after adding disulfiram/copper complex (DSF/Cu) to triple-negative breast cancer cells, the intracellular iron levels, lipid ROS, and malondialdehyde are significantly increased, while the level of glutathione is significantly decreased. These are all important markers of ferroptosis. It has also been found that DSF/Cu boosts lipid peroxidation, which gives rise to a steep rise in the activity of heme oxygenase-1 (HO-1). As a result, it triggers the death of triple-negative breast cancer (TNBC) cells via ferroptosis. These research results suggest that DSF/Cu could serve as a prospective drug treatment approach for triple-negative breast cancer along with other tumors [19]. Nuclear factor erythroid 2-related factor 2 (Nrf2) belongs to the redox transcriptionally active factors in eukaryotic cells and has functions such as stress protection and anti-aging. It assumes an essential role in biological undertakings such as antioxidation and anti-inflammation. Heme oxygenase-1, a key element in certain biological pathways, is a downstream target gene transcriptionally governed by Nrf2 and is also an important antioxidant molecule [20]. Zhao Y et al. discovered that DSF/Cu is capable of elevating the level of intracellular free iron ions, boosting lipid peroxidation, and finally triggering ferroptosis. Suppressing Nrf2 or HO-1 can boost the susceptibility of oral squamous cell carcinoma (OSCC) cells to DSF/Cu-triggered ferroptosis. It has also been confirmed in an in vivo xenograft tumor model that DSF/Cu restrains the progression of OSCC via curbing the expression of Nrf2/HO-1, thereby presenting a novel approach for treating OSCC [21] (Fig. 2).

On the other hand, copper ions are capable of triggering ferroptosis by modulating the signaling pathways associated with ferroptosis. GPX4 functions as a pivotal negative regulatory factor in the process of ferroptosis. It is capable of precluding the initiation of ferroptosis via the elimination of phospholipid hydroperoxides. Xue Q et al. found that copper boosts the occurrence of ferroptosis in pancreatic cancer cells via instigating the autophagous breakdown of the GPX4 protein. Mechanistically, it was found that copper directly attaches to cysteines C107 and C148 within the GPX4 protein, elevates the ubiquitination level of GPX4, promotes the generation of GPX4 aggregates, and further induces the degradation of the GPX4 protein and the occurrence of ferroptosis through the autophagy receptor TAX1BP1 [22]. Daihong Cai et al. reported that CuGluc, as a soluble divalent copper complex, can induce ferroptosis by boosting the accumulation of lipid peroxidation and inhibiting the activity of GPX4, and remarkably restricts tumor growth in vivo, thus providing a crucial strategy for the development of highly effective antitumor copper complexes [23]. In addition, Wei G et al. found that elesclomol, a carrier of copper ions, can expedite the breakdown of the ATPase copper transporting alpha (ATP7A). After combined treatment with elesclomol and copper ions, copper ions are retained in mitochondria, reactive oxygen species accumulate, and then the breakdown of SLC7A11 is promoted, additionally promoting the redox imbalance and ferroptosis of colorectal cancer cells, revealing that elesclomol exerts an antitumor effect in colorectal cancer via precisely aiming at the ATP7A/SLC7A11/ferroptosis signal [24]. The DSF/Cu can exert antitumor activity in nasopharyngeal carcinoma cells through the ferroptosis pathways mediated by ROS/MAPK and p53, while the ROS scavenger N-acetyl-L-cysteine (NAC) has the ability to regulate the concentrations of reactive oxygen species within nasopharyngeal carcinoma cells and the lipid levels. Combined treatment with cisplatin can synergistically increase the antitumor effect in DSF/Cu xenograft tumors in vivo [25]. The molecular mechanism underlying this synergistic effect can be further elucidated through copper transporter 1 (CTR1). Schoeberl et al. found that cisplatin co-accumulates with CTR1 in cancer cells, and the expression of CTR1 is regulated by copper, thereby mediating the intracellular transport of both copper and cisplatin [26]. Cisplatin-induced accumulation of CTR1 enhances DSF/Cu-mediated copper influx, promoting copper overload-induced cuproptosis [6]. Meanwhile, cisplatin and copper synergistically increase ROS levels, depleting glutathione and inhibiting GPX4, which aggravates ferroptosis and results in an additive oxidative stress effect [27]. These research results indicate that the ferroptosis pathways mediated by copper ions exert a vital function in the origination, evolution of tumors and the generation of drug resistance. Targeting the Cu/ferroptosis pathways may become an important molecular strategy for tumor treatment and drug sensitization. At the molecular level, copper ions further regulate ferroptosis by affecting mitochondrial function and the antioxidant system. For example, copper is a cofactor of cytochrome c oxidase (COX), and the impairment of COX function can trigger the over-accumulation of ROS and cell death [28, 29]. Fan Li et al. discovered that copper depletion can restrain the protein expression of GPX4. Consequently, through mitochondrial disruption and antioxidant pathways, cells become more sensitive to the ferroptosis inducer Erastin. This implies that the depletion of copper ions is conducive to the occurrence of ferroptosis [30]. Therefore, the regulation of ferroptosis by copper ions is also a process of homeostatic regulation. Both copper ion overload and depletion may affect the occurrence of ferroptosis through multiple mechanisms (Fig. 2a).

#### Iron - ion overload can induce cuproptosis

Ferroptosis is an iron-dependent type of lipid peroxidation accumulation, so iron overload can induce ferroptosis. However, recent studies have indicated that other programmed cell death modalities, including cuproptosis, often accompany the process of ferroptosis. Ferroptosis-inducing agents like sorafenib and erastin can boost ferroptosis by promoting the aggregation of copper-dependent lipoylated proteins in primary liver cancer cells. Mechanistically, sorafenib and erastin can elevate protein lipoylation. They do this by curbing the degradation of ferredoxin 1 (FDX1) protein, which is mediated by mitochondrial matrix-related proteases. Moreover, they cut down on the synthesis of intracellular copper–GSH complex through inhibiting cystine uptake. Therefore, the combined employment of agents that induce ferroptosis and copper ionophores, so as to target both ferroptosis and cuproptosis, may well serve as a novel tactic in the treatment of primary liver cancer [31]. As cuproptosis was only introduced as a novel cell death type in 2022, the related studies on the influence of iron ions and ferroptosis on cuproptosis are very limited. However, we believe that iron ions and ferroptosis are very likely to be involved in copper ion-mediated cuproptosis through multiple mechanisms (Fig. 2b).

## The mutual regulation between molecules related to cuproptosis and ferroptosis in the two death pathways

### Cuproptosis-associated molecules affect ferroptosis

**Fig. 3:**
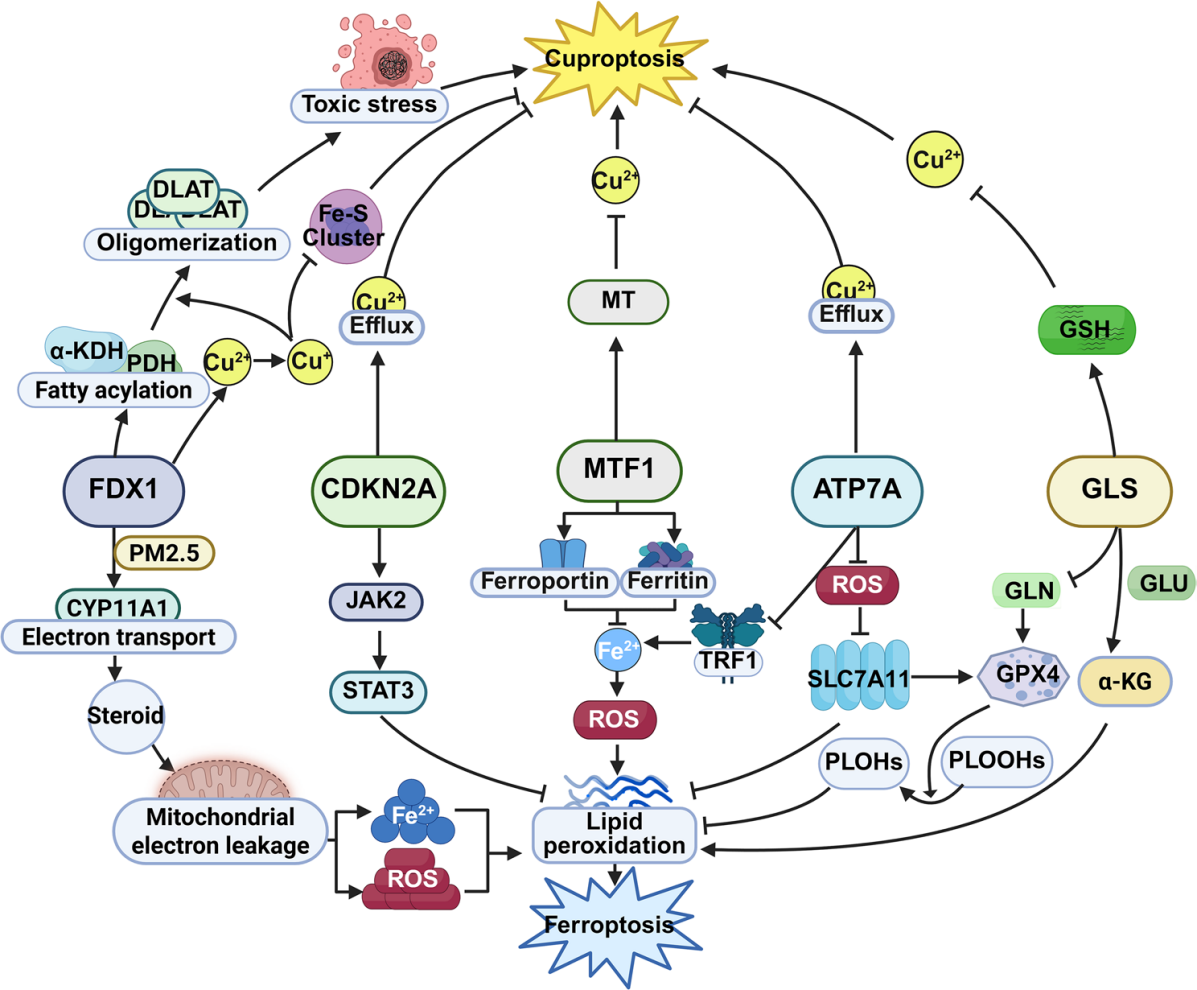
Mechanisms by which cuproptosis-related genes regulate both ferroptosis and cuproptosis. (Created with Biorender.com)

Many molecules have been reported to be involved in the process of cuproptosis, including FDX1, glutaminase (GLS), ATP7A, cyclin-dependent kinase inhibitor 2 A (CDKN2A), metal-regulatory transcription factor 1 (MTF1) (Fig. 3). These molecules play critical roles in copper ion homeostasis and the relevant signaling pathways of cuproptosis. In recent years, an increasing number of studies have shown that these cuproptosis-related molecules are involved in the process of ferroptosis.

#### FDX1

Studies have found that FDX1, a type of ferredoxin, can promote the occurrence of cuproptosis in multiple ways. When intracellular copper ions pile up excessively, the iron-sulfur protein encoded by FDX1 has the capacity to reduce Cu²⁺ to Cu⁺. This reduction inhibits the synthesis of iron-sulfur (Fe-S) cluster which is relevant to mitochondrial respiration. As a result, proteotoxic stress is induced, which eventually leads to cell death. Moreover, FDX1, acting as an upstream regulator of protein lipoylation, promotes the lipoylation of pyruvate dehydrogenase (PDH) and α-ketoglutarate dehydrogenase (αKDH). Cu⁺ binds to their lipoyl groups, resulting in the oligomerization of dihydrolipoamide S-acetyltransferase (DLAT), triggering proteotoxic stress and finally resulting in cell death [6]. Therefore, FDX1 is a key regulatory molecule for the occurrence of cuproptosis (Fig. 3).

Recent studies have further revealed that exposure to particulate matter with an aerodynamic diameter ≤ 2.5 μm (PM2.5) can induce the upregulation of FDX1 in the mouse testicular interstitial cell line TM-3, which enhances electron transfer to cytochrome P450 family 11 subfamily A member 1 (CYP11A1) and promotes steroidogenesis. The increased steroidogenic activity elevates mitochondrial electron leakage, thereby leading to higher levels of reactive oxygen species (ROS) and ferrous ion overload in testicular interstitial cells. These alterations jointly initiate the auto-oxidation of PUFAs in the cell membrane, resulting in excessive lipid peroxide accumulation and the onset of ferroptosis. This suggests that FDX1 is an important regulatory factor for PM2.5-induced ferroptosis in testicular interstitial cells [32] (Fig. 3). In addition, knocking down FDX1 expression has been found to increase mitochondrial membrane potential loss and lipid peroxidation in ovarian cancer cells treated with cisplatin, thereby enhancing ferroptosis and increasing cisplatin sensitivity. This indicates that FDX1 normally inhibits cisplatin-induced ferroptosis in ovarian cancer cells, thereby promoting cisplatin resistance [33]. These findings suggest that FDX1 has dual roles in the regulation of ferroptosis, either promoting or inhibiting it. This paradoxical phenomenon might be explained by three factors: (1) tissue/cell-type differences, since variations in mitochondrial metabolism, content of Fe–S clusters, and antioxidant networks (e.g., GSH/GPX4, Nrf2 signaling) can alter the downstream effects of FDX1; (2) dose–time dependence, as acute high-dose stress (e.g., copper overload) may directly trigger mitochondrial protein lipoylation and copper toxicity to promote lipid peroxidation, whereas chronic low-dose stress may induce metabolic reprogramming and iron redistribution, producing opposite outcomes; and (3) cancer type/drug interactions, since the combined effects of chemotherapeutics (such as cisplatin) and copper metabolism may reshape cell death pathways, making FDX1 knockdown in certain cancers instead enhance drug-induced ferroptosis.

Taken together, FDX1 not only directly governs the occurrence of cuproptosis through copper metabolism, but also regulates ferroptosis by modulating homeostasis of Fe–S proteins, mitochondrial function, and ROS metabolism. Its opposite effects (promotion or inhibition of ferroptosis) in different cellular contexts highlight FDX1 as a potential molecular link between cuproptosis and ferroptosis. In other words, FDX1 is not only a central regulator of cuproptosis but may also act as a dual role mediator in the cross-regulatory network of metal ion homeostasis and lipid peroxidation.

#### GLS

GLS catalyzes the conversion of GLN(glutamine) to GLU and is a key enzyme in cellular GLN metabolism. Glutamate serves as an essential precursor for GSH synthesis. Once synthesized, GSH functions as a critical intracellular copper-chelating and reducing agent that can bind copper ions and thereby suppress copper-induced cuproptosis. Therefore, GLS mainly affects the occurrence of cuproptosis by influencing the level of GSH, a substance capable of binding copper ions within cells [6].

In ferroptosis, GLU derived from GLS participates in two major metabolic pathways closely associated with this process: (1) as a precursor for GSH biosynthesis, GLU contributes to GSH production, which in turn acts as a cofactor for GPX4. GPX4 facilitates the reduction of phospholipid hydroperoxides (PLOOHs) to their corresponding alcohols (phospholipid alcohols), thereby inhibiting ferroptosis; and (2) GLU can be converted into α-ketoglutarate (α-KG), which enters the TCA cycle to support mitochondrial metabolism and redox flux and promote the generation of oxidation-sensitive lipid precursors. Under conditions of cystine/system xc⁻ limitation, α-KG derived from GLN catabolism enhances lipid peroxidation, thereby promoting ferroptosis. Consequently, the effect of GLS on ferroptosis is context-dependent, determined by the maintenance of intracellular GSH synthesis, the level of GLS, and the overall metabolic and redox state of the cell [34, 35] (Fig. 3).

#### ATP7A

ATP7A encodes a transmembrane copper-transporting P-type ATPase and serves as a central regulator of cellular copper homeostasis. It is primarily localized to the Golgi apparatus but can be redistributed to the plasma membrane under copper overload or specific stimuli, where it facilitates the efflux of excess intracellular copper and delivers copper to the secretory pathway for the maturation of cuproenzymes, thereby maintaining copper balance. This transport activity reduces abnormal copper retention in mitochondria and other subcellular compartments, thereby suppressing copper overload–induced cuproptosis. In addition, disruption of copper homeostasis can secondarily perturb iron metabolism and promote ferroptosis through enhanced oxidative stress.

In ATP7A-deficient zebrafish models, studies have reported a significant increase in iron content within the central nervous system, accompanied by abnormal expression of iron metabolism–related proteins, such as transferrin receptor 1 (TFR1) and ferritin. The accumulation of an unstable intracellular labile iron pool can aggravate ROS production and lead to decreased GPX4 expression or enzymatic activity, thereby weakening the clearance of lipid peroxides and enhancing ferroptosis sensitivity [36]. Moreover, in colorectal cancer cells, treatment with the copper ionophore Elesclomol results in reduced ATP7A levels and increased ubiquitination, which impairs copper efflux, promotes mitochondrial copper accumulation, and causes ROS overflow. Excess ROS decreases the protein stability of SLC7A11—a key transporter for GSH synthesis—whose dysfunction leads to insufficient GSH production, further reducing GPX4 activity and ultimately triggering ferroptosis through the accumulation of lipid peroxidation products [24] (Fig. 3).

Therefore, ATP7A acts as a critical crossroad between two metal-dependent cell death pathways: on the one hand, it directly prevents cuproptosis by limiting mitochondrial copper retention; on the other hand, its role in maintaining copper homeostasis prevents secondary disturbances in iron metabolism and ROS overflow, thereby sustaining the SLC7A11, GSH, and GPX4 antioxidant defense axis and reducing ferroptosis susceptibility. Loss of ATP7A function not only causes copper accumulation and proteotoxic stress, but also amplifies lipid peroxidation reactions, suggesting that ATP7A status may serve as a potential reference indicator for therapeutic interventions targeting metal-dependent cell death (Fig. 3).

#### CDKN2A

CDKN2A functions as a tumor suppressor gene encoding the p16 protein along with the p14 protein. The p16 protein serves to inhibit CDK4 and CDK6, preventing cells from advancing from the G1 phase to the S phase, ultimately suppressing cell proliferation [37]. In the investigation carried out by Tsvetkov et al., with the application of CRISPR/Cas9 technology screening, it was confirmed that CDKN2A is an important negative regulatory factor for cuproptosis and is capable of inhibiting the occurrence of cuproptosis [6]. Meanwhile, numerous results of bioinformatics analysis studies have also verified that CDKN2A is an important cuproptosis-related gene and diagnostic marker in various tumors [38–40]. Mechanistically, Cheng X et al. reported that CDKN2A suppresses cuproptosis by enhancing glycolysis and regulating copper homeostasis (including promoting copper efflux), thereby reducing mitochondrial copper accumulation and TCA protein lipoylation [41]. With respect to the relationship between CDKN2A and ferroptosis, studies have shown that loss of CDKN2A in GBM leads to remodeling of lipid metabolism, with oxidizable PUFAs redistributed into distinct lipid compartments. This results in elevated lipid peroxidation and renders tumor cells more susceptible to ferroptosis [42]. Taken together, these findings indicate that CDKN2A establishes a potential link between the two metal-dependent cell death pathways through the regulation of metabolism and redox homeostasis. Specifically, when CDKN2A loss leads to simultaneous dysregulation of copper homeostasis and lipid metabolism, mitochondrial copper retention–induced ROS and PUFA-enrichment–induced lipid peroxidation may mutually amplify one another, thereby forming a positive feedback loop between cuproptosis and ferroptosis. This dual role highlights CDKN2A not only as a crossroads of metal-dependent cell death but also as a critical determinant of tumor drug resistance and prognosis (Fig. 3).

#### MTF1

MTF1 drives the transcription of downstream metallothionein (MT) genes by recognizing metal response elements (MREs) in promoter regions. Upregulation of MT enhances the buffering and detoxification capacity of cells against excess copper, thereby reducing the accumulation of free copper in subcellular compartments (especially in mitochondria) and decreasing the occurrence of cuproptosis [43].

Chen et al. reported that in the human breast cancer cell line MDA-MB-231, inhibition of the DNA damage response kinase ATM promotes the nuclear translocation of MTF1. Nuclear MTF1 can upregulate the expression of ferritin and the iron exporter FPN1, thereby lowering the labile iron pool, reducing iron-dependent lipid peroxidation, and preventing ferroptosis [44]. Taken together, when MTF1 function remains intact, it can attenuate both copper-and iron-mediated oxidative damage as well as the induction of the two forms of cell death. Conversely, impaired function or transcriptional downregulation of MTF1 may simultaneously exacerbate copper accumulation and iron pool instability, promote ROS generation and membrane lipid peroxidation, and render cells more susceptible to cuproptosis and ferroptosis. Therefore, assessing the expression level and activity status of MTF1 may serve as a biomarker for predicting responsiveness to metal-dependent therapies. Future strategies may involve combining MTF1 inhibitors with cuproptosis and ferroptosis inducers or stratifying patients according to tumor MTF1 status, thereby overcoming drug resistance and improving therapeutic efficacy (Fig. 3).

### The impact of ferroptosis-related molecules on cuproptosis

Currently, most of the research has centered on the independent working mechanisms of ferroptosis and cuproptosis. Comparatively speaking, there have been relatively scant investigations into how the regulatory molecules of ferroptosis influence cuproptosis. Theoretically, if the regulatory molecules of ferroptosis affect the balance of intracellular metal ions or the redox state, they may indirectly influence the occurrence of cuproptosis. For example, a decline in the activity of GPX4 is likely to trigger a rise in oxidative stress. This augmented oxidative stress, in turn, could potentially disrupt the metabolism and equilibrium of copper ions. For instance, by influencing the homeostasis of the antioxidant GSH, copper ions may not be maintained in an appropriate oxidation state, thus affecting the metabolic pathways in which they are involved and further influencing cuproptosis [45]. However, the extent of such an impact and the specific mechanisms remain unclear at present and require further research to explore.

## Signal pathways that are jointly involved in ferroptosis and cuproptosis

### The p53 signaling pathway is jointly involved in both ferroptosis and cuproptosis

**Fig. 4:**
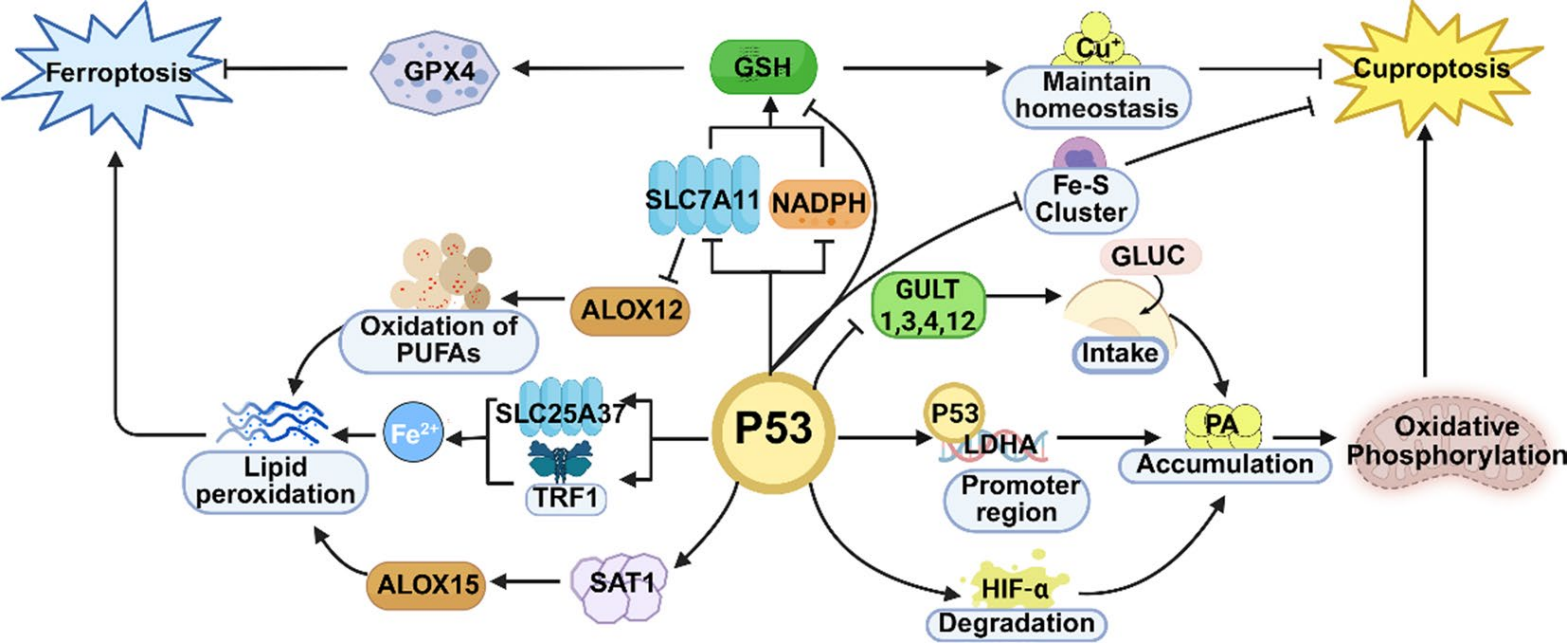
The p53 signal transduction pathway is involved in the mechanisms of both ferroptosis and cuproptosis. (Created with Biorender.com )

p53 functions as a significant tumor suppressor protein and an essential transcription factor. By toggling the expression of some crucial genes between activation and inhibition, it is capable of taking part in multiple biological processes, namely the cell cycle, apoptosis, differentiation, senescence, growth arrest, and DNA damage repair. The p53 signal transduction pathway is a crucial regulatory pathway for cell survival and death, and its chief role lies in safeguarding cells against injuries caused by DNA damage or other internal and external stimulating factors [46]. Under normal circumstances, the p53 protein is in an inhibited state due to the ubiquitination and degradation mediated by murine double minute 2 (MDM2) [47, 48], ARF binding protein 1 (ARF-BP1) [49], constitutive photomorphogenic 1 (COP1) [50], C terminus of Hsc70-interacting protein (CHIP) [51] and p53-Induced RING-H2 Protein (PIRH2) [52, 53]. However, upon exposure to cellular stress, kinases such as vaccinia-related kinase 1 (VRK1) [54, 55], Aurora kinase A (Aurora-A) [56] can phosphorylate p53, thereby preventing MDM2-mediated degradation. In this way, it can prevent cell proliferation and start apoptosis via prompting cell cycle arrest, DNA repair, or the production of antioxidant proteins. More and more studies in recent years have shown that the p53 signal pathway assumes a crucial part in both ferroptosis and cuproptosis (Fig. 4).

#### The p53 signaling pathway is involved in ferroptosis

SLC7A11, as the functional subunit of the system Xc⁻, maintains GPX4 activity by regulating cystine uptake and GSH synthesis, thereby suppressing ferroptosis. This dual process is conducive to GSH biosynthesis. By potentiating the activity of the membrane lipid repair enzyme, GPX4, it effectively thwarts the onset of ferroptosis instigated by lipid peroxidation at the cellular level. As an essential transcription factor, p53 is capable of docking onto the promoter region of SLC7A11, thereby exerting an inhibitory effect on its transcriptional activity and subsequent protein expression. Through the SLC7A11/GSH/GPX4 signaling cascade, this regulatory mechanism fosters ferroptosis within cells and concomitantly restrains the malignant progression of tumors, as evidenced by references [56, 57].

Similarly, extant studies have corroborated that p53 can also modulate the ferroptosis pathway via an alternative route, independent of the SLC7A11/GSH/GPX4 axis. Arachidonate lipoxygenase 12 (ALOX12), a lipid oxidase, occupies a central position in the ferroptosis regulatory network. SLC7A11 can form a direct physical association with ALOX12, thereby confining its enzymatic function. Hence, when p53 instigates the downregulation of SLC7A11, ALOX12 is released concurrently. The unshackled ALOX12 then proceeds to oxidize the polyunsaturated fatty acid chains present in cell membrane phospholipids, ultimately precipitating ferroptosis within cells [58, 59]. Likewise, p53 can upregulate the expression of spermidine/spermine N1-acetyltransferase 1 (SAT1). Activation of SAT1, in turn, serves to augment the function of arachidonate lipoxygenase 15 (ALOX15), another pivotal member of the ALOX family. This coordination intensifies the ferroptosis process within cells [60, 61].

At the level of iron metabolism, p53 can also regulate the distribution of mitochondrial and intracellular iron. Studies have found that p53 can target and regulate the transcriptional activity and expression of SLC25A37. Via this regulatory process, it expedites the absorption of iron ions. Subsequently, this leads to an overload of iron ions in mitochondria and induces the onset of ferroptosis at the cellular level [62]. p53 is also capable of homing in on and regulating the expression of TFR1. By doing so, it boosts the uptake of iron ions, ultimately giving rise to ferroptosis in cells [63]. Furthermore, cytoglobin is capable of enhancing the sensitivity of colon cancer cells to ferroptosis by activating the p53/Yes-associated protein 1 (YAP1) signaling pathway [64] (Fig. 4).

In summary, p53 promotes ferroptosis not only by suppressing the antioxidant axis (SLC7A11/GSH/GPX4) but also by activating lipid oxidases and altering iron metabolism, thereby exerting multiple effects.

#### The p53 signaling pathway is involved in cuproptosis

p53 is of great significance in reshaping tumor metabolism, covering the modulation of glycolysis along with oxidative phosphorylation. Given that these two intricately linked metabolic processes are highly relevant to the sensitivity of cells to cuproptosis, p53 is likely to play a substantial regulatory role in this process. Especially in cells where oxidative phosphorylation is the main energy acquisition mode, p53 inhibits glucose metabolism through different mechanisms. It can downregulate the expression or functional activity of glucose transporters, such as GLUT1, GLUT3, and GLUT4, reducing glucose intake [65–67]. Moreover, through direct binding to the promoter region of lactate dehydrogenase A (LDHA) [68] or facilitating the breakdown of HIF-1α [69], which is a transcription factor of LDHA, p53 causes the accumulation of pyruvate in cells, thus driving the metabolic shift towards the citric acid cycle and aerobic phosphorylation, and further facilitating the occurrence of cuproptosis. Meanwhile, p53 reduces the level of GSH by inhibiting the expression of SLC7A11 and suppressing the production of nicotinamide adenine dinucleotide phosphate (NADPH) [70, 71], thereby affecting the intracellular copper ion homeostasis and promoting cuproptosis [6]. In addition, the onset of cuproptosis goes hand in hand with the breakdown of Fe-S cluster proteins. Fe-S cluster proteins are a prevalent class of proteins that are extensively engaged as cofactors in numerous biological processes, including enzymatic catalysis, electron transfer, and coping with metabolic stress. p53 may also promote the occurrence of cuproptosis by disrupting the stability of Fe-S cluster proteins and reducing the level of glutathione, which is an intracellular copper ion chelator [72, 73].

### The Nrf2 signaling pathway participates in both ferroptosis and cuproptosis

**Fig. 5:**
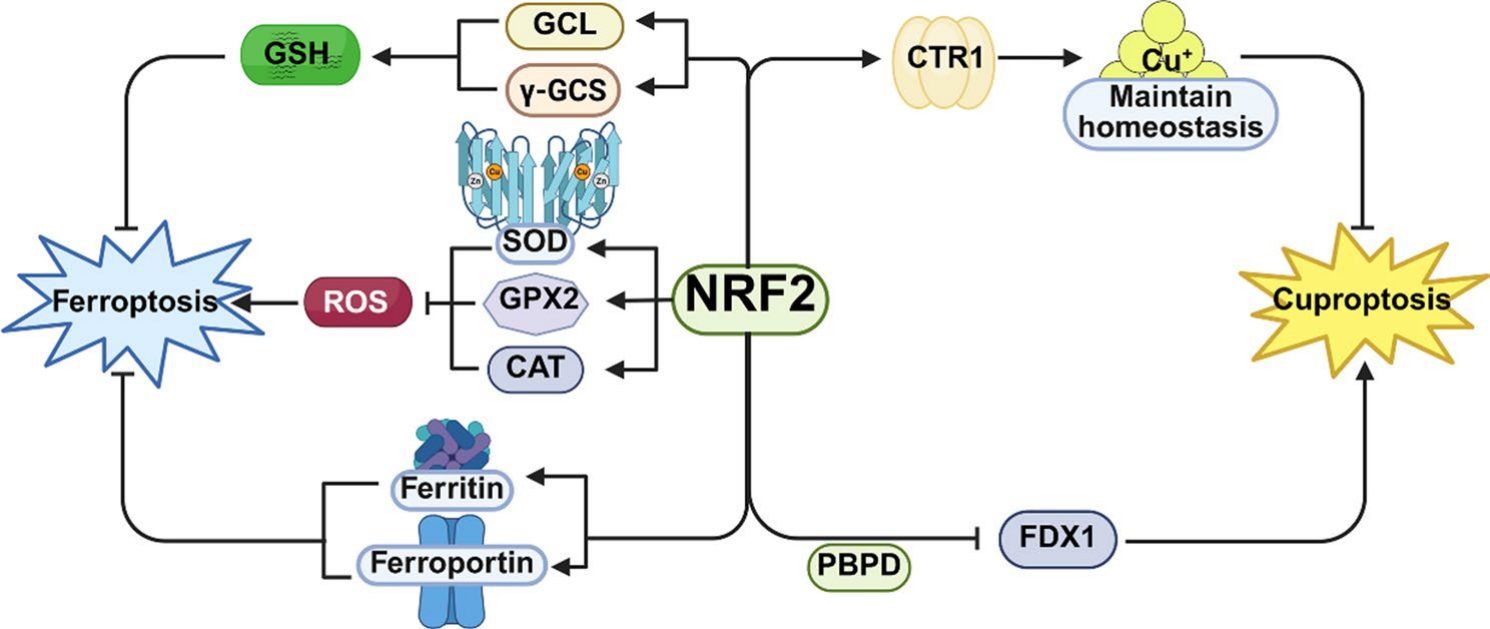
The Nrf2 signaling pathway participates in both ferroptosis and cuproptosis. (Created with Biorender.com )

Nrf2, a transcription factor, is of crucial significance for the oxidative stress response. It attaches to the antioxidant-response element (ARE) within the gene promoter region, thus playing a pivotal role in regulating the transcription and expression of antioxidant-protective genes [74]. Under normal conditions, the BCR (KEAP1) and cullin 3 (CUL3) complex can promote the ubiquitination and breakdown of Nrf2 through the ubiquitin-proteasome pathway [75, 76]; however, during an oxidative stress response including exposure to toxins and reactive oxygen species (ROS), oncogenic signals, gene mutations, autophagy interference, and metabolic changes, electrophilic metabolites will suppress the function of the BCR complex, facilitate the formation of a heterodimer between NFE2L2/Nrf2 and a type of small Maf protein, cause their accumulation within cell nucleus, and interact with other transcription factors and co-factors to regulate the transcriptional regulation of destination genes [77], thus playing a crucial part in cellular oxidative stress and tumor progression (Fig. 5).

#### The role of the Nrf2 signaling pathway in ferroptosis

The production of ROS constitutes a crucial hallmark of ferroptosis. Nrf2 can boost the transcriptional function and the expression levels of antioxidant enzymes including glutathione peroxidase 2 (GPX2), superoxide dismutase (SOD), and catalase (CAT) by regulating their ARE, thereby scavenging intracellular ROS and reducing the level of intracellular oxidative stress [78–81] (Fig. 5). Meanwhile, GSH is the substrate of GPX4. With GSH at its disposal, it can turn lipid peroxides into non-toxic lipid alcohols, which in turn averts the accumulation of lipid peroxidation and inhibits the emergence of ferroptosis. Nrf2 can promote the biosynthesis of GSH by enhancing the transcriptional activity and expression of GLU-cysteine ligase (GCL) and γ-glutamyl cysteine synthetase (γ-GCS), thereby halting the onset of ferroptosis [82]. In addition, iron is a key participant in the Fenton reaction during the progression of ferroptosis. Iron ions can promote the formation of intracellular reactive oxygen species and lipid peroxidation, thus facilitating ferroptosis. Furthermore, Nrf2 has been confirmed to promote iron efflux by enhancing the manifestation of ferritin and ferroportin, thereby reducing the intracellular iron level and suppressing the onset of ferroptosis [83–85]. In tumor cells, the Nrf2 signaling pathway is often abnormally activated. On the one hand, this activation enables tumor cells to acquire the ability to resist ferroptosis, which helps tumor cells survive and proliferate in a harsh microenvironment. For example, in some drug-resistant tumor cells, the elevated Nrf2 maintains antioxidant activity, iron homeostasis and avoids ferroptosis induced by chemotherapeutic drugs. On the other hand, suppressing the Nrf2 signaling pathway can also amplify the sensitivity of tumor cells to ferroptosis inducers, thereby improving the efficacy of tumor treatment [86, 87] (Fig. 5).

#### The role of the Nrf2 signaling pathway in cuproptosis

During the process of cuproptosis, high levels of ROS will be generated in cells. The initiation of the Nrf2 pathway may enhance the antioxidant capacity of cells and alleviate the damage to cells caused by ROS to a certain extent, thus inhibiting cuproptosis. For example, the copper-quinone-glucose oxidase nanoparticles with self-destructive and multi-enzyme activities (abbreviated as CQG NPs) designed by Luying Qiao can inhibit the Nrf2 and NQO1 signaling pathway to disrupt the antioxidant defense machinery of tumor cells and induce cuproptosis in tumor cells, suggesting that the Nrf2 signaling pathway holds a key position in cuproptosis-related tumor treatment [88]. Meanwhile, Nrf2 may affect the expression or activity of proteins related to intracellular copper metabolism, thereby playing a significant role in the governance of cuproptosis. As an example, Nrf2 has the ability to regulate the expression of CTR1 in hepatocytes, thereby enhancing the cells’ capacity to uptake copper ions. When the intracellular copper ion concentration spikes to an excessive value, the initiation of the Nrf2 pathway is capable of regulating the expression of copper-transporting proteins, enabling cells to adjust their uptake, transport, and excretion processes according to changes in the concentration of copper ions to maintain copper ion homeostasis [89]. At the same time, a derivative of curcumin, 1-propyl-3,5-bis(2-bromobenzylidene)-4-piperidinone (PBPD), has been confirmed to significantly inhibit the proliferation of cervical cancer cells and increase cuproptosis in cells. Mechanistically, studies have shown that PBPD can potently suppress the Nrf2 signaling pathway and elevate the expression of FDX1. Through this, it fosters cuproptosis in cells and restrains the malignant advancement of cervical cancer. Evidently, Nrf2-mediated cuproptosis is of great significance in the malignant development of cervical cancer [90] (Fig. 5).

## Ferroptosis and cuproptosis are both implicated in the tumor malignant progression and treatment resistance

Ferroptosis and cuproptosis each represent types of programmed cell death. The ability to resist death marks an outstanding biological attribute of tumors. They can respectively provide survival advantages for tumor cells by influencing tumor cells, tumor microenvironment, and tumor stemness characteristics, thus facilitating the onset and progression of tumors, as well as the emergence of treatment resistance.

### The role of ferroptosis in tumors

Ferroptosis is a type of regulated cell death that is iron-dependent. It is triggered by the build-up of excessive lipid peroxides on the cell membrane. This build-up results in the impairment of the membrane’s integrity, ultimately inducing cell death. Under normal circumstances, the inducers and defense factors of ferroptosis within cells are in a dynamic balance, thereby maintaining the intactness of the cell membrane and cell survival. In tumor cells, when iron ions are overloaded and the intracellular antioxidant system is inhibited, tumor cells will undergo ferroptosis due to the build-up of excessive lipid peroxides on the membrane, resulting in the inhibition of tumor growth; conversely, if the defense mechanisms against ferroptosis dominate, tumor cells will exhibit resistance to death, thereby leading to tumor progression and metastasis [91]. It can be seen that the induction and defense regulation of ferroptosis exert a significant influence on the malignant advancement of tumors.

The induction of ferroptosis is primarily centered on the initiation of lipid peroxidation, driven by factors such as dysregulated iron uptake, aberrant polyunsaturated fatty acid metabolism, and excessive ROS accumulation, all of which act as direct triggers of ferroptosis in tumor cells.

Tumor cells usually have a relatively high iron uptake ability and promote the intake of iron ions by regulating the production of iron transport proteins such as TFR1. Meanwhile, the breakdown of the iron storage protein ferritin will also release more iron ions, increasing the intracellular iron ion concentration and providing the necessary conditions for ferroptosis in tumor cells [92, 93]. The abnormal metabolic process of unsaturated fatty acids in tumor cells gives rise to a growth in lipid peroxidation, thereby prompting ferroptosis among tumor cells. For example, the upregulated expression of acyl-CoA synthetase long-chain family member 4 (ACSL4) can promote the synthesis and esterification of unsaturated fatty acids, increase the content of unsaturated fatty acids on the cell membrane, and promote lipid peroxidation reactions on the cell membrane, thereby inhibiting the progression of hepatocellular carcinoma and enhancing therapeutic sensitivity [94]. Similarly, increased intracellular ROS production can also promote lipid peroxidation, thereby inducing ferroptosis [95]. The sources of ROS include the mitochondrial respiratory chain, NADPH oxidase, xanthine oxidase, and external environmental stimuli, which collectively induce ferroptosis in cells [96, 97].

The mechanism by which tumor cells resist ferroptosis centers on “antioxidant defense,” wherein core pathways and factors cooperate with each other to form an integrated defense network. Among them, the SLC7A11/GSH/GPX4 axis is the most critical defense pathway, allowing tumor cells to evade immune- and drug-induced lipid peroxidation–mediated cell death [98]. At the same time, ferroptosis suppressor protein 1 (FSP1), as a GSH-independent defense factor, can reduce coenzyme Q (CoQ) to ubiquinol (CoQH2) at the plasma membrane. CoQH2 functions as a lipophilic radical-trapping agent, directly blocking the initiation of lipid peroxidation [99]. Glutathione S-transferase P1 (GSTP1) serves as a supplementary mechanism, possessing both glutathione transferase activity and selenium-independent glutathione peroxidase activity. It can catalyze the conjugation of GSH with the lipid peroxidation product 4-hydroxynonenal (4-HNE) to neutralize toxicity, and directly eliminate lipid peroxides [100]. Dihydroorotate dehydrogenase (DHODH), on the other hand, contributes to mitochondrial defense by reducing coenzyme Q (ubiquinone) to its reduced form (ubiquinol), thereby inhibiting lipid peroxidation in the inner mitochondrial membrane and limiting local ROS accumulation, which provides additional protection for tumor cells [99]. Meanwhile, in hepatocellular carcinoma cells, sorafenib can induce ferroptosis, but tumors activate an MT-1G/NRF2-dependent program to upregulate antioxidant genes such as SLC7A11 and GPX4, thereby suppressing lipid peroxidation and developing acquired resistance [101]. Excessive activation of these defense mechanisms disrupts the dynamic balance between “ferroptosis induction” and “defense” in tumor cells, leading to various tumor types to develop tolerance to ferroptosis inducers, ultimately contributing to malignant progression and therapeutic resistance [102–104].

**Fig. 6:**
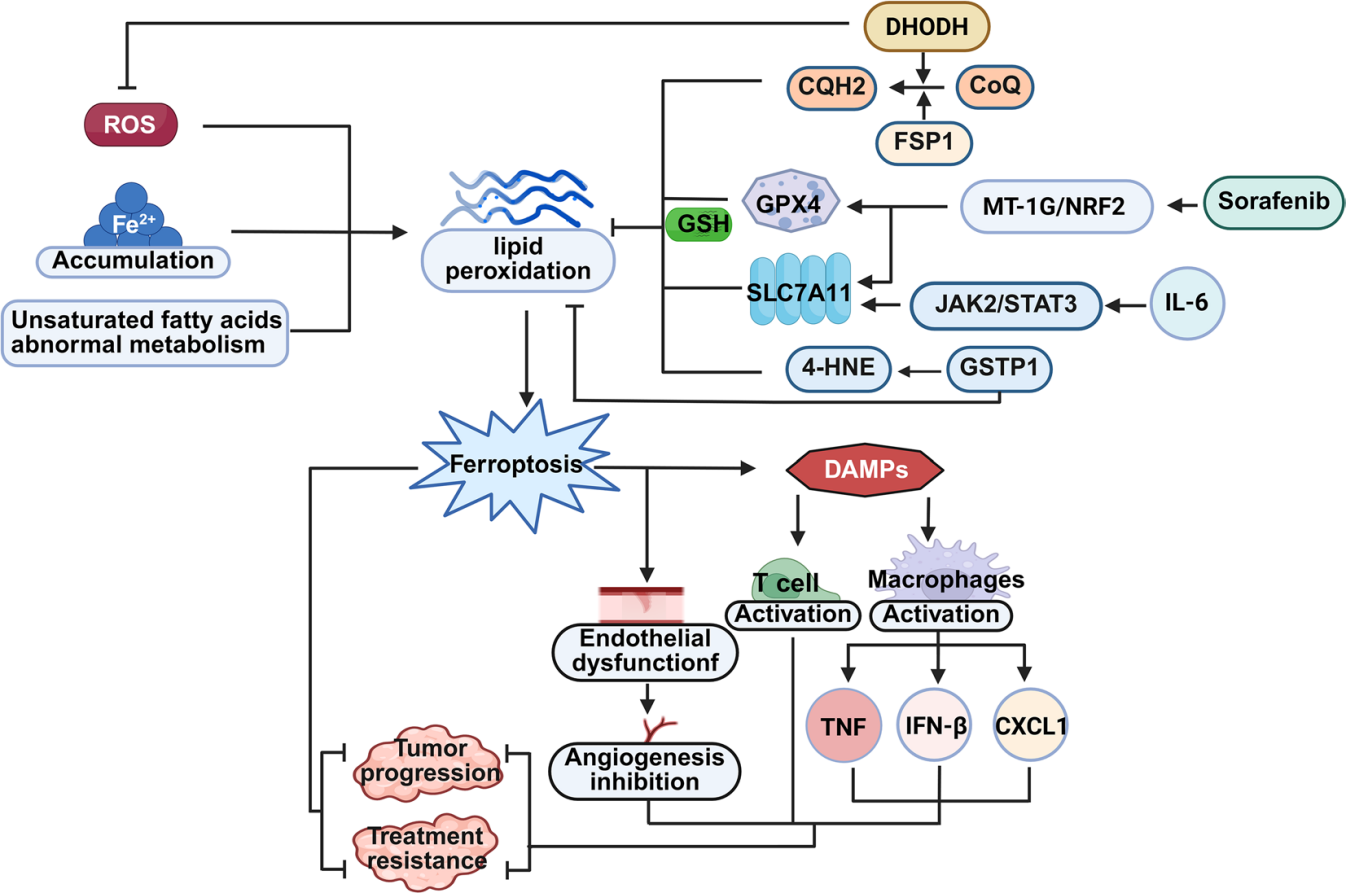
The role of ferroptosis in tumors. (Created with Biorender.com)

The tumor microenvironment (TME) is a critical region for interactions between tumor cells and adjacent normal tissues. It is composed of tumor cells, tumor-associated fibroblasts, immune cells, secreted products, and non-cellular components of the extracellular matrix [104], whose regulation of ferroptosis exhibits a “bidirectional effect.” On the one hand, when tumor cells undergo ferroptosis, they release damage-associated molecular patterns (DAMPs), which not only enhance anti-tumor immune responses by activating tumor-associated antigen presentation and T-cell activity, but also activate macrophages to secrete pro-inflammatory cytokines (such as TNF, IFN-β, and CXCL1 [105]), thereby enhancing phagocytic capacity and anti-tumor activity [106]. In addition, metabolites of ferroptosis can damage vascular endothelial cell membranes, leading to endothelial dysfunction and subsequently suppressing tumor angiogenesis [107]. These processes strengthen anti-tumor effects and increase drug sensitivity through the dual mechanisms of “direct tumor suppression and microenvironmental regulation.” On the other hand, the inflammatory microenvironment can provide tumor cells with additional resistance advantages. For example, in head and neck squamous cell carcinoma, tumor-associated cytokine interleukin-6 (IL-6) can activate the JAK2/STAT3 pathway to transcriptionally upregulate SLC7A11 (xCT), thereby enhancing tumor cell resistance to ferroptosis inducers such as Erastin [108]. This further reinforces ferroptosis resistance in tumor cells and diminishes therapeutic efficacy (Fig. 6).

### The role of cuproptosis in tumors

Cuproptosis is initiated by copper ion overload, lipoylation of mitochondrial proteins, subsequent protein oligomerization, and destabilization of iron–sulfur clusters, ultimately causing proteotoxic stress in tumor cells. During tumorigenesis and tumor progression, the regulation of copper ion homeostasis is of great significance for both the development of tumors and the emergence of treatment resistance. On the one hand, copper concentrations in the tumor tissues and sera of many cancer patients are significantly elevated, which is closely associated with the increased demand for copper in tumor cells (compared with non-dividing cells, tumor cells exhibit a markedly higher requirement for copper [109]). Copper can bind to and activate pro-angiogenic and growth-related signaling pathways, including vascular endothelial growth factor (VEGF) [110], fibroblast growth factor-2 (FGF-2) [111], interleukin-8 (IL-8) [112], and interleukin-1β (IL-1β), thereby further contributing to tumorigenesis, angiogenesis, invasion, and metastasis [113]. On the other hand, copper ion overload can bind to mitochondrial lipoylated proteins, inducing protein oligomerization and destabilization of Fe–S clusters, which leads to cell death and thereby exerts antitumor effects. Therefore, abnormalities in copper ion homeostasis and protein lipoylation modification represent important factors by which cuproptosis participates in tumorigenesis and malignant progression.

Although research on the mechanisms of resistance to cuproptosis is still limited, existing studies have revealed that changes in the expression of regulatory proteins can affect the efficacy of cuproptosis. In hepatocellular carcinoma cells, the serine/threonine protein kinase MELK not only enhances the monomer levels of the key cuproptosis gene DLAT by activating the PI3K/mTOR pathway and improves mitochondrial function, thereby promoting resistance to elesclomol and supporting tumor cell survival and progression. Moreover, it downregulates the cytotoxic effects of elesclomol via this pathway, directly contributing to drug resistance [114]. In addition, research on cuproptosis-related genes has demonstrated that cuproptosis-suppressing genes such as CDKN2A are closely associated with tumor progression and therapeutic resistance. In multiple myeloma cells, high CDKN2A expression simultaneously inhibits immune infiltration and downregulates the core cuproptosis gene FDX1, thereby weakening both antitumor immune responses and tumor sensitivity to cuproptosis. Conversely, inhibition of CDKN2A significantly reduces tumor cell resistance, suggesting that its high expression may be directly associated with an increased risk of therapeutic resistance in patients [115]. These findings indicate that tumor cells can attenuate cuproptosis and acquire resistance by regulating key signaling pathways and the expression of cuproptosis-related genes.

**Fig. 7:**
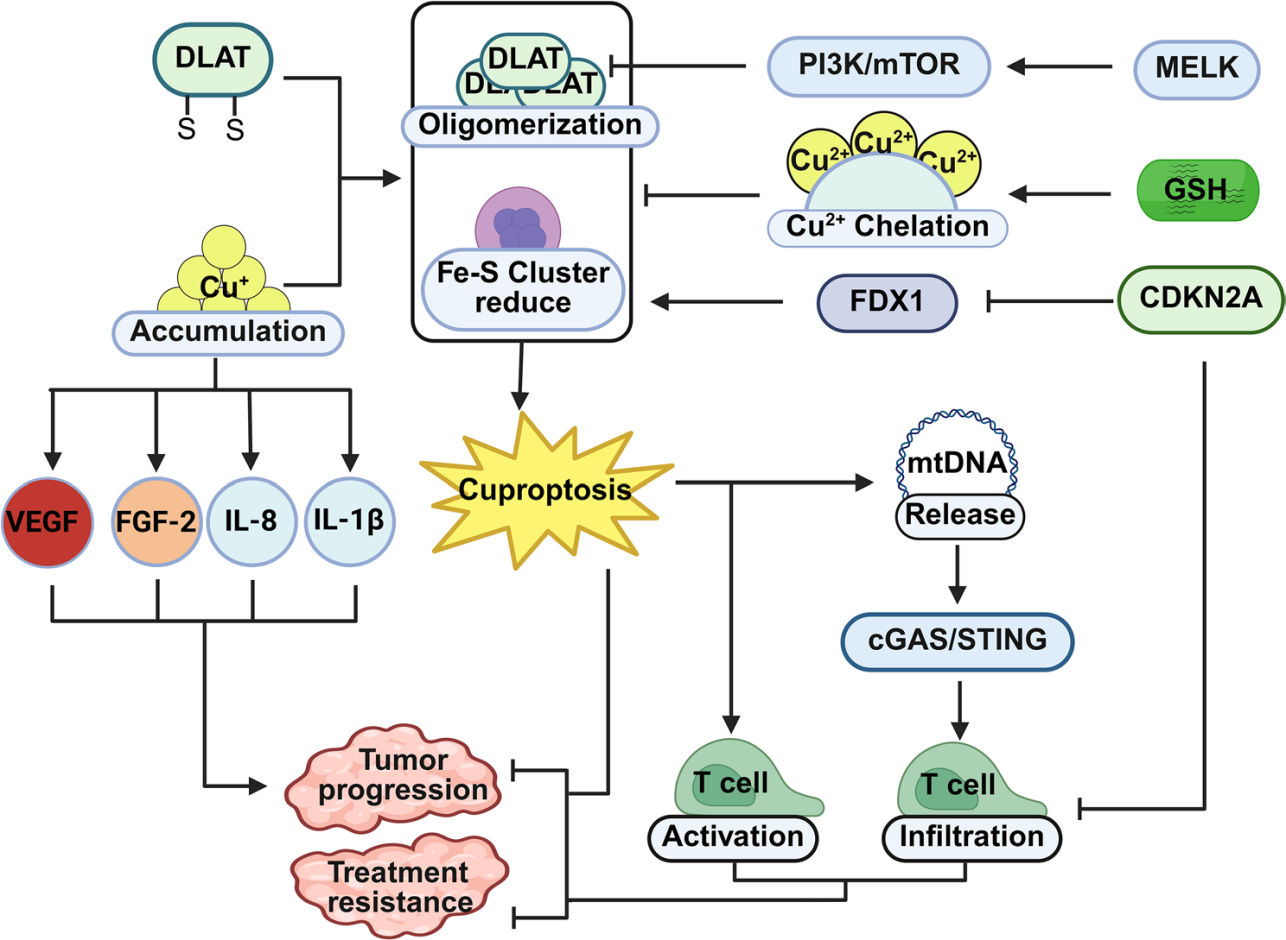
The role of cuproptosis in tumors. (Created with Biorender.com)

In addition, cuproptosis is closely associated with the tumor microenvironment. Multiple pan-cancer bioinformatics and clinical sample analyses have demonstrated that the expression of cuproptosis-related genes (such as FDX1 and SLC31A1) is significantly correlated with the level of CD8⁺ T cell infiltration, the proportion of immunosuppressive cell populations, and the sensitivity to immune checkpoint inhibitors and chemotherapeutic agents in tumors, suggesting that copper metabolic status can reshape TME by altering cell death patterns and immune cell composition [116, 117]. Mechanistically, cuproptosis induced by Cu-DPPZ-Py⁺ or Cu-elesclomol can trigger mitochondrial damage and promote the release of mitochondrial DNA (mtDNA). The leaked mtDNA, acting as a DAMP, is recognized by cytoplasmic cGAS, thereby activating the STING pathway and inducing the release of type I interferons, and also proinflammatory cytokines such as TNF-α and IFN-γ. This process facilitates dendritic cell maturation as well as the recruitment and activation of CD8⁺ T cells, ultimately enhancing tumor immunogenicity and antitumor immune responses [118] (Fig. 7).

### Ferroptosis- and cuproptosis-targeted therapies and clinical translational value

Targeted therapies for ferroptosis mainly focus on regulating iron homeostasis and lipid peroxidation to enhance tumor cell sensitivity. Multiple candidate drugs, technical approaches, and therapeutic strategies have already been developed, with accumulating evidence from preclinical to early clinical stages. On one hand, several ferroptosis inducers have been developed. For example, erastin and its analogs can inhibit system Xc⁻, thereby reducing cystine uptake, leading to insufficient GSH synthesis and ferroptosis induction [7]. RSL3 and its derivatives directly inhibit GPX4 activity, blocking the reduction of lipid peroxides [119]. In addition, nanotechnology has been widely applied in ferroptosis-targeted therapies. For instance, nanomaterials based on ferrite or metal–organic frameworks (MOFs) can release Fe²⁺ in the tumor microenvironment, promote Fenton reactions, and amplify ROS levels, thereby significantly enhancing ferroptotic effects [120]. On the other hand, targeting key signaling pathways such as p53 and Nrf2 has also been shown to modulate tumor cell sensitivity to ferroptosis, providing new intervention points for combination therapies [121].

Cuproptosis, as a newly proposed form of programmed cell death, has therapeutic strategies primarily centered on disrupting copper ion homeostasis and inhibiting mitochondrial function. The most representative drug is elesclomol, a small-molecule copper ionophore that delivers copper ions into mitochondria, induces oxidative stress and mitochondrial dysfunction, and thereby triggers cell death. Clinical studies have shown that elesclomol has entered early clinical trials in multiple cancers. In the phase III SYMMETRY trial, elesclomol combined with paclitaxel was evaluated in advanced melanoma patients but did not significantly improve overall survival in the entire cohort. However, subgroup analysis revealed that patients with normal baseline lactate dehydrogenase (LDH) levels benefited substantially from elesclomol treatment, suggesting that its efficacy may depend on the metabolic background of patients [122].

**Fig. 8:**
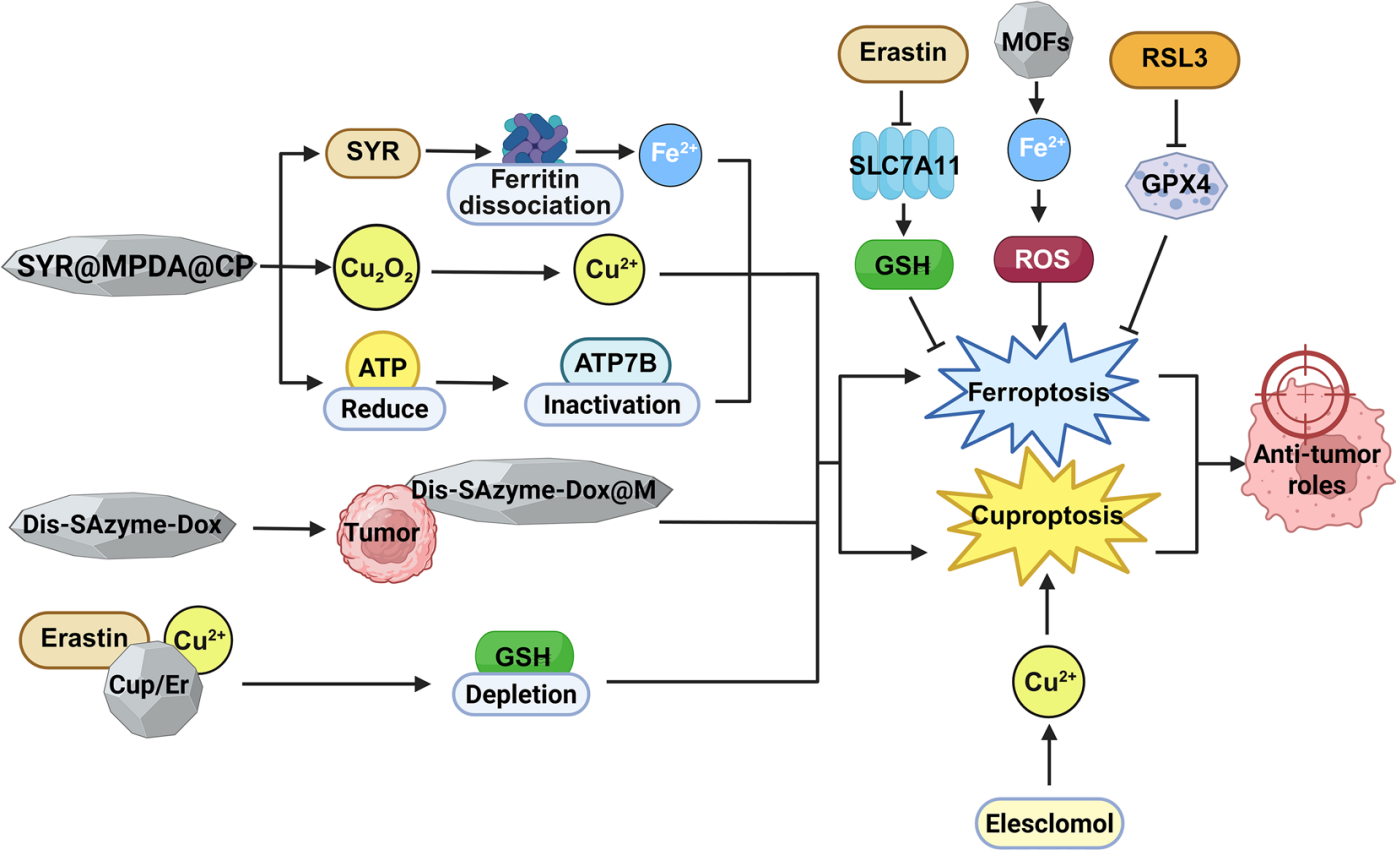
Ferroptosis- and cuproptosis-targeted therapies. (Created with Biorender.com)

Understanding the crosstalk between ferroptosis and cuproptosis helps in designing combination therapies to overcome drug resistance. However, single-target therapies are prone to triggering compensatory mechanisms in tumors, leading to diminished efficacy over time. In cuproptosis-targeted therapies, tumors may upregulate copper efflux pumps (e.g., ATP7A/ATP7B) to enhance copper export, reduce intracellular copper accumulation, and consequently develop resistance [123]. Leveraging this, several studies have proposed “dual-targeting” strategies that simultaneously activate ferroptosis and cuproptosis. One pioneering study by Zhi H et al. designed a mesoporous polydopamine (MPDA)-based nanocarrier system Syrosingopine (SYR)@MPDA@Cu₂O₂ (CP) that could specifically release SYR in breast cancer models. SYR inhibited lactate efflux, leading to intracellular lactate accumulation and acidification, which on one hand promoted the dissociation of ferritin to release endogenous iron, and on the other hand suppressed glycolysis and reduced ATP levels, thereby inactivating ATP7B. Together with Cu²⁺ released from CP, this system amplified intracellular Fe²⁺/Cu²⁺ levels, ultimately co-activating ferroptosis and cuproptosis and enhancing immunogenic cell death [124]. One recent innovative approach involves engineered nanocarriers. For example, in a murine hepatocellular carcinoma model, a diselenide-bridged diatomic nanozyme loaded with doxorubicin (dis-SAzyme-Dox), camouflaged with tumor cell membranes (dis-SAzyme-Dox@M), was able to specifically accumulate in tumor tissues and simultaneously induce ferroptosis and cuproptosis in tumor cells. This process also released intracellular antigens and inflammatory cytokines, thereby activating dendritic cells and significantly enhancing immunogenic cell death [125]. Similarly, researchers synthesized CuP/Er nanoparticles that co-loaded copper ions and the ferroptosis inducer erastin. These nanoparticles depleted intracellular GSH, inhibited the Warburg effect, enhanced lipid peroxidation, and achieved synergistic activation of cuproptosis and ferroptosis. Notably, CuP/Er not only directly triggered immunogenic cell death in tumor cells but also upregulated PD-L1 expression and enhanced T-cell infiltration, leading to strong synergistic effects with immune checkpoint inhibitors, which markedly suppressed the growth of colorectal cancer and triple-negative breast cancer [126] (Fig. 8). These combinational strategies demonstrate that dual targeting of metal ion–related cell death pathways can not only directly enhance tumor cell death but also improve the efficacy of immunotherapy by increasing tumor immunogenicity.

However, due to tumor heterogeneity, not all patients can benefit from the above strategies, making patient stratification an essential consideration for clinical translation. In recent years, prognostic and stratification models based on ferroptosis- and cuproptosis-related molecules (including specific lncRNAs or gene sets) have demonstrated significant predictive value in various cancer types. For instance, a cuproptosis-related lncRNA signature (AC009053.3, AC017071.1, MFF-DT, RNF213-AS1, AC091588.1, AL118556.1, Z68871.1, AL451123.1) has been constructed and shown to predict prognosis in triple-negative breast cancer, among which Z68871.1 may serve as a promising therapeutic target [127]. Similarly, in lung adenocarcinoma, a risk model based on ferroptosis-related lncRNAs (AL606489.1, AC106047.1, LINC02081, AC090559.1, AC026355.1, FAM83A-AS1, AL034397.3, AC092171.5, AC010980.2, AC123595.1) exhibited strong prognostic value and provided a reliable basis for patient risk stratification [128]. Ultimately, integrating both ferroptosis- and cuproptosis-related features will be key to realizing precision medicine. Indeed, a combined risk model incorporating BACH1, CDCA3, and TIMP1 has been developed to predict prognosis in low-grade gliomas, while also offering mechanistic insights and new directions for immunotherapy [129]. These risk models and molecular subtyping strategies may enable precise patient stratification, effectively addressing the challenges posed by tumor heterogeneity and providing a rational basis for identifying subgroups most likely to benefit from specific targeted therapies.

Despite the promising outlook of dual-target strategies, several major challenges remain for clinical translation. First is the issue of systemic toxicity and safety management—although copper ionophores such as Elesclomol exhibit relatively low toxicity toward normal tissues [130, 131], systemic regulation of copper and iron still poses potential risks: copper overload may induce oxidative stress and neurotoxicity [132], while iron dysregulation can lead to anemia as well as liver and cardiac injury [133]. Thus, tumor-specific delivery systems (e.g., tumor microenvironment-responsive nanocarriers, engineered antibody–drug conjugates), combined with dynamic monitoring of serum copper and iron levels, are needed to optimize dosing and minimize normal tissue damage. Second are the technical bottlenecks of drug delivery systems—metal ion carriers often suffer from poor tumor-targeting efficiency and are prone to nonspecific uptake by normal tissues [134]; in addition, drug release from nanocarriers is significantly influenced by variations in the tumor microenvironment (e.g., pH, enzymatic activity), leading to uneven release profiles. This may result in excessively high local drug concentrations with toxicity or insufficient levels that compromise efficacy [135], posing a challenge for the co-delivery of copper ions and ferroptosis inducers. Third, there is a gap in integrated diagnostic-therapeutic approaches—few studies have designed “early diagnosis + combined therapy” strategies based on the shared regulatory mechanisms of ferroptosis and cuproptosis. Exploiting metabolic crosstalk, such as mitochondrial function regulation, to link diagnostic biomarkers with therapeutic targets remains a critical frontier. Finally, the mechanistic understanding of cuproptosis is still incomplete, with open questions regarding its detailed pathways, tumor subtype–specific effects, and interplay with ferroptosis at the signaling level, all of which require further validation through high-quality studies.

Taken together, current strategies have already demonstrated multiple feasible approaches for simultaneously targeting ferroptosis and cuproptosis. However, for broad and safe clinical application, breakthroughs are still needed in precise patient stratification, controllable and tumor-specific drug delivery systems, and the development of low-toxicity therapeutic strategies.

## Conclusions and prospects

Cuproptosis and ferroptosis constitute two novel forms of programmed cell death induced by metal ion overload discovered in recent years. The mitochondrion is the common site where they occur. Both of them lead to an elevation of intracellular peroxides and reactive oxygen species due to mitochondrial stress, thereby resulting in cell death. This review summarizes the regulation of intracellular metal ion homeostasis, the occurrence processes, the signaling pathways of ferroptosis and cuproptosis, as well as the possible mutual influences during the emergence and advancement of tumors, which is of certain significance for better clarifying the principles governing tumorigenesis and tumor progression and for clinical diagnosis and treatment.

Although this review focuses on the commonalities and interconnections between cuproptosis and ferroptosis, the two are fundamentally distinct modes of regulated cell death. In terms of initiation mechanisms, cuproptosis is triggered by copper ion overload, whereas ferroptosis depends on the participation of iron ions. Regarding core molecular pathways, cuproptosis and ferroptosis are primarily governed by the FDX1/DLAT axis and the SLC7A11/GPX4 axis, respectively. Therefore, future research should adhere to the principle of “seeking common ground while preserving differences”: on one hand, to explore their synergistic regulatory mechanisms and provide a foundation for developing combination therapeutic strategies; on the other hand, to clarify their unique characteristics and enable the precise design of single-target interventions. Both directions are expected to provide important insights for elucidating tumorigenesis mechanisms and developing novel diagnostic and therapeutic technologies. However, research on cuproptosis is still in its infancy, and its detailed mechanisms remain to be further investigated, including the intracellular transport of copper ions, the substrate types and regulation of lipoylation modifications, and the molecular events that trigger cuproptosis, all of which remain unclear. Moreover, the roles of key molecules involved in cuproptosis within the ferroptosis pathway are not yet well understood, leaving the mechanisms by which cuproptosis drives ferroptosis largely unexplored. In terms of drug development, most current efforts have focused on individual targets within either cuproptosis or ferroptosis, with limited progress in identifying shared targets and developing drugs that act on them. Additionally, given the toxicity and off-target effects of existing compounds and the inherent heterogeneity of tumors, achieving efficient targeted drug delivery and personalized treatment strategies remains a critical challenge and research priority for the future. In summary, in-depth elucidation of the mechanisms underlying cuproptosis and the advancement of its coordinated application with ferroptosis will constitute key directions for future research in this field.

## Data Availability

No datasets were generated or analysed during the current study.

## Abbreviations

ACSL4: Acyl-CoA synthetase long chain family member 4
ALOX12: Arachidonate 12-lipoxygenase
ALOX15: Arachidonate 15-lipoxygenase
ARF-BP1: ARF binding protein 1
ARE: Antioxidant-response element
ATM: Ataxia-telangiectasia mutated
ATP7A: ATPase copper transporting alpha
Aurora-A: Aurora kinase A
BCR: BTB-Cullin3-RING E3 Ubiquitin Ligase Complex
CAT: Catalase
CDKN2A: Cyclin-dependent kinase inhibitor 2 A
CHIP: C terminus of Hsc70-interacting protein
COP1: Constitutive photomorphogenic 1
CoQ: Coenzyme Q
CoQH2: Reduced coenzyme Q
CP: Cu₂O₂
COX: Cyclooxygenase
CQG NPs: Copper -Quinone - Glucose Oxidase Nanoparticles
CUL3: Cullin 3
CYP11A1: Cytochrome P450 family 11 subfamily A member 1
DAMPS: Damage-Associated Molecular Patterns
DHODH: Dihydroorotate dehydrogenase
DLAT: Dihydrolipoamide S-acetyltransferase
DSF: Disulfiram
FDX1: Ferredoxin 1
Fe-S: Iron-Sulfur
FPN1: Ferroportin 1
GBM: Glioma Multiforme
GCL: Glutamate-cysteine ligase
GLS: Glutaminase
GLU: Glutamate
GPX2: Glutathione peroxidase 2
GPX4: Glutathione peroxidase 4
GSTP1: Glutathione S-transferase Pi 1
GSH: Glutathione
HO-1: Heme oxygenase-1
IL-1β: Interleukin-1
IL-6: Interleukin-6
IL-8: Interleukin-8
KEAP1: Kelch-like ECH-associated protein 1
LDHA: Lactate dehydrogenase A
MDM2: Murine Double Minute 2
MELK: Maternal embryonic leucine zipper kinase
MOFs: Metal–organic frameworks
MPDA: Mesoporous polydopamine
MREs: Metal response elements
MT: Metallothionein
MTF1: Metal-regulatory transcription factor 1
mtDNA: Mitochondrial DNA
NADPH: Nicotinamide adenine dinucleotide phosphate
NAC: N-acetyl-L-cysteine
NQO1: NAD(P)H: quinone oxidoreductase 1
Nrf2: Nuclear factor erythroid 2-related factor 2
OSCC: Oral squamous cell carcinoma
PBPD: 1-propyl-3,5-bis(2-bromobenzylidene)-4-piperidinone
PDH: Pyruvate dehydrogenase
PIRH2: p53-Induced RING-H2 Protein
PLOOHs: Phospholipid hydroperoxides
PUFAs: Polyunsaturated fatty acids
PM2.5: Particulate matter ≤ 2.5 μm
RCC: Renal cell carcinomas
ROS: Reactive oxygen species
SAT1: Spermidine/Spermine N1-acetyltransferase 1
SLC3A2: Solute carrier family 3 member 2
SLC7A11: Solute carrier family 7 member 11
SOD: Superoxide dismutase
SYR: Syrosingopine
System Xc⁻: Cystine/GLUtamate antiporter
TCA: Tricarboxylic acid cycle
TFR1: Transferrin receptor 1
TNBC: Triple-negative breast cancer
TME: Tumor microenvironment
VRK1: Vaccinia-related Kinase 1
VEGF: Vascular endothelial growth factor
FGF-2: Fibroblast growth factor-2
YAP1: Yes-associated protein 1
αKDH: α- ketoglutarate dehydrogenase
α-KG: α-ketoglutarate
γ-GCS: γ-glutamylcysteine synthetase
4-HNE: 4-Hydroxy-2-nonenal
